# Parkinson’s disease and translational research

**DOI:** 10.1186/s40035-020-00223-0

**Published:** 2020-12-01

**Authors:** Elisabeth Dinter, Theodora Saridaki, Leonie Diederichs, Heinz Reichmann, Björn H. Falkenburger

**Affiliations:** 1 Department of Neurology, Technische Universität Dresden, Dresden, Germany; 2 Deutsches Zentrum für Neurodegenerative Erkrankungen, Dresden, Germany; 3 Department of Neurology, RWTH University Aachen, Aachen, Germany

**Keywords:** α-Synuclein, Pre-formed fibrils, Protein aggregates, Aggresome, Dopamine deficiency, Medium spiny neurons, Autophagy

## Abstract

Parkinson’s disease (PD) is diagnosed when patients exhibit bradykinesia with tremor and/or rigidity, and when these symptoms respond to dopaminergic medications. Yet in the last years there was a greater recognition of additional aspects of the disease including non-motor symptoms and prodromal states with associated pathology in various regions of the nervous system. In this review we discuss current concepts of two major alterations found during the course of the disease: cytoplasmic aggregates of the protein α-synuclein and the degeneration of dopaminergic neurons. We provide an overview of new approaches in this field based on current concepts and latest literature. In many areas, translational research on PD has advanced the understanding of the disease but there is still a need for more effective therapeutic options based on the insights into the basic biological phenomena.

## Introduction to Parkinson’s disease (PD)

PD is diagnosed when patients exhibit bradykinesia with tremor and/or rigidity, and when these symptoms respond to dopaminergic medications [1]. In the past few years, additional aspects of the disease including non-motor symptoms and prodromal states with associated pathology in various regions of the nervous system gained increasing attention. The non-motor symptoms affect the quality of life because they cannot be treated as well as the typical motor symptoms, thus constituting one of the most important therapeutic challenges in PD therapy. The other challenge lies in the management of motor fluctuations, i.e. the PD motor symptoms remain responsive to dopaminergic medications but the mobility is tightly linked to serum concentrations, which requires short dosing-intervals or the use of medication pumps. In the brains of PD patients, we have found two major pathologies, cytoplasmic aggregates of alpha-synuclein (aSyn) and degeneration of dopaminergic neurons. The current therapies and translational research are focusing on these two aspects, which will be discussed in the following.

## Part 1: Synuclein pathology

In 1997, we began to know that rare familial forms of PD could be caused by point mutations in the aSyn gene *SNCA* [2], including the mutations of A53T, A30P, E46K, H50Q and G51D, and duplication of the *SNCA* locus. In addition, polymorphisms in the *SNCA* locus are a risk factor for sporadic PD [3, 4]. Following the detection of PD-associated mutations, aSyn was identified as a major constituent of Lewy bodies (LB) [5]. These cytosolic inclusions of aggregated proteins were first described histologically by Fritz Heinrich Lewy in 1912 and associated with PD by Konstantin Nikolaevich Trétiakoff in 1919 [6]. Almost a century later, Heiko Braak described the distribution of LB in the brain and suggested that the aSyn pathology spreads along the axonal projections [7]. According to this concept, the aSyn pathology starts in the periphery and enters the brain through the olfactory bulb or along the vagal nerve [8]. Then it spreads transsynaptically to the limbic system respectively to further brainstem nuclei including the substantia nigra pars compacta. Subsequently, the pathology spreads to the neocortical areas [9].

**Fig. 1:**
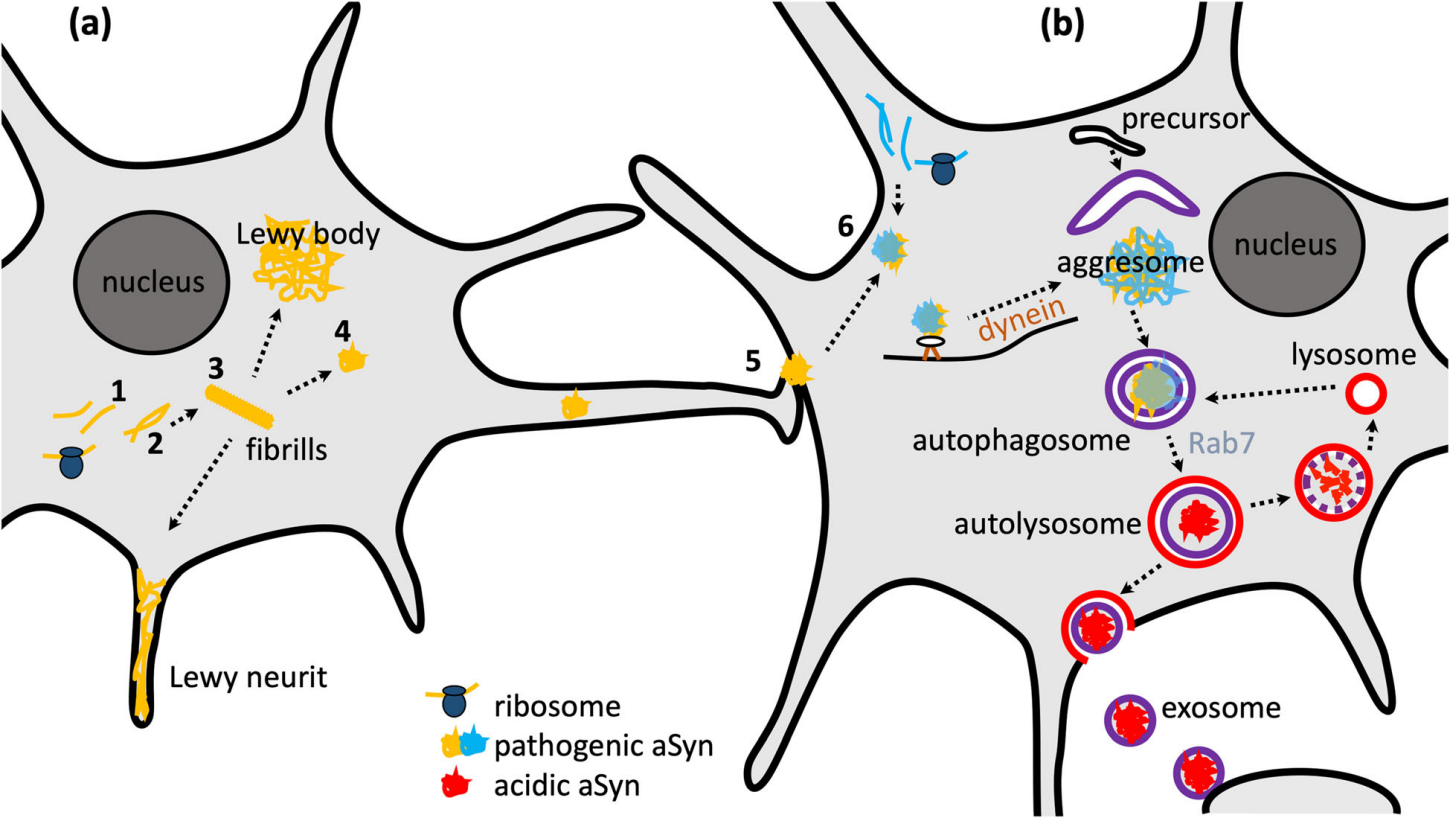
Aggregation, transport and clearance of α-synuclein. **a** Concept of aggregation and spreading: After ribosomal translation of pathogenic α-synuclein (aSyn), monomers (1) form oligomers (2) and primary nucleation with formation of the first aggregate takes place. Subsequent steps are fibril elongation (3) and secondary nucleation with formation of further nuclei, e.g. by fibrils breaking (4). The aggregates are transported along the axonal projections, secreted and taken up by a neighboring cell (5). The aggregation of aSyn monomers is greatly enhanced by addition of even small quantities of aggregates, which serve as nuclei and replace the slow step of primary nucleation by the faster step of secondary nucleation. This process is called seeding (6). **b** Transport and autophagic clearance of aSyn: Aggregates are dynein-dependently transported to the perinuclear region to form aggresomes. Parts of the cytosol containing aggregates get engulfed by a membrane to form autophagosomes. Subsequently, Rab7 regulates the trafficking of autophagosomal and lysosomal vesicles and their fusion towards autolysosomes, followed by degradation of the vesicle content. There is also evidence for the secretion via exosomal release

Lewy neurites are swollen neurites that contain aSyn filaments [10] and in fact incorporate the majority of aSyn aggregates [11]. Dystrophic aSyn-positive neurites have also been observed in the peripheral nervous system [12]. In addition to PD, other disorders are also associated with aSyn aggregates, including dementia with Lewy bodies and multisystem atrophy (MSA). In MSA, aSyn aggregates are located in glial rather than in neuronal cells [13]. The emergence and spread of the aSyn pathology are illustrated in Fig. 1a.

### Clinical correlates of synuclein pathology

The concept of the spreading aSyn pathology has convinced clinicians because it is consistent with the fact that PD motor symptoms are accompanied by and often preceded by non-motor symptoms. This concept also led to the definition of prodromal or pre-motor stage of PD [14]. Early non-motor symptoms include hyposmia [15] – plausibly caused by aSyn pathology in the olfactory bulb - and gastrointestinal symptoms [16] – plausibly caused by aSyn pathology in the vagus nerve. The rapid-eye-movement sleep behavioral disorder is caused by dysfunction of a specific brainstem nucleus and is one of the most specific predictors of PD [17]. The most important non-motor symptom of advanced PD is dementia, which is plausibly caused by cortical aSyn pathology [18].

It should be noted, however, that the correlation between the distribution of Lewy bodies in the brain and clinical symptoms is not perfect. There are patients with “incidental” Lewy bodies that clinically do not differ from age-matched controls and patients with an “atypical” distribution pattern of aSyn pathology [19]. Furthermore, even some familial forms of PD lack Lewy bodies [20]. Some authors have argued that the clinical symptoms are better explained by the distribution of Lewy neurites than by Lewy bodies given that the former likely produce greater functional impairment [21].

The non-motor symptoms are prominent features of PD and constitute a major impact on quality of life [22]. This can be attributed to the fact that classical motor symptoms respond well to dopaminergic medication, whereas symptomatically, the non-motor symptoms often cannot be treated well.

### Synuclein aggregation and spreading

aSyn aggregation has been studied extensively in vitro, using biophysical methods to assess different steps of the aggregation process and measure different aSyn species [23]. Steps of aggregation include primary nucleation (i.e. formation of the first aggregate from monomeric aSyn), fibril elongation/aggregate growth, and secondary nucleation (formation of further nuclei, e.g. by fibrils breaking) (Fig. 1a). Small molecules can inhibit the aggregation by interfering with any of these steps [24, 25]. We have recently demonstrated that an engineered β-wrappin can bind to the αSyn monomer and prevent aSyn aggregation in a substoichiometric manner [23]. Aggregation of aSyn monomer is greatly enhanced by addition of even small quantities of aggregates, which serve as nuclei and replace the slow step of primary nucleation by the faster step of secondary nucleation. This process is called seeding (step 6 in Fig. 1).

The spread of aSyn pathology through the brain can therefore be decomposed into three distinct processes: (i) the transport of aSyn aggregates along the axonal projections to new brain areas, (ii) the secretion and uptake of aSyn aggregates and (iii) seeding (Fig. 1). The transport was initially described as mainly retrograde, but both directions have been observed in cellular and mouse models [26, 27]. The mechanisms of secretion and uptake remain to be clarified, however, the exosomal release and endocytosis have been proposed to be involved [28–30]. Some evidence has suggested that the aSyn secretion is dependent on the presynaptic activity [31–33] and the lysosomal processing may also be involved in the transmission [34, 35]. The endogenous synuclein and a fibrillary structure of aSyn are not essential for the spread of aSyn through the brain [36, 37], and environmental toxins can trigger aSyn aggregation [8].

Although aSyn fibrils consisting largely of beta-sheets are considered the major constituents of Lewy bodies and Lewy neurites, the beta-sheet formation is not necessary for aSyn toxicity [38, 39]. One of the most controversial questions about aSyn aggregation is whether different synucleinopathies, specifically PD and MSA, are characterized and/or caused by different “strains” of aSyn aggregates [40]. A recent refinement of the “protein misfolding cyclic amplification” method has indicated that PD and MSA are indeed characterized by different strains of aSyn aggregates and that their differences can be detected even in cerebrospinal fluid from PD and MSA patients [41]. Also in mouse models, different pathologies have been observed following injection of “PD” and “MSA” seeds [42, 43].

### Aggregate clearance and functional significance of Lewy bodies

Cells do not remain passive to the formation of aSyn aggregates. Using fluorescently tagged aSyn and live-cell microscopy we observed active transport of aSyn aggregates towards a perinuclear region where aggregates accumulate [44]. This region is called aggresome and is located at the microtubule organizing center [45]. The transport of aggregates towards the aggresome is mediated by microtubules and dynein motors (Fig. 1b). Aggregates are recruited to dynein motors by different adaptor proteins, including p62, heat-shock proteins and HDAC6 [46]. Ubiquitination is a typical first step for the recognition of aggregates by this system, but it is neither necessary nor required [47].

**Fig. 2:**
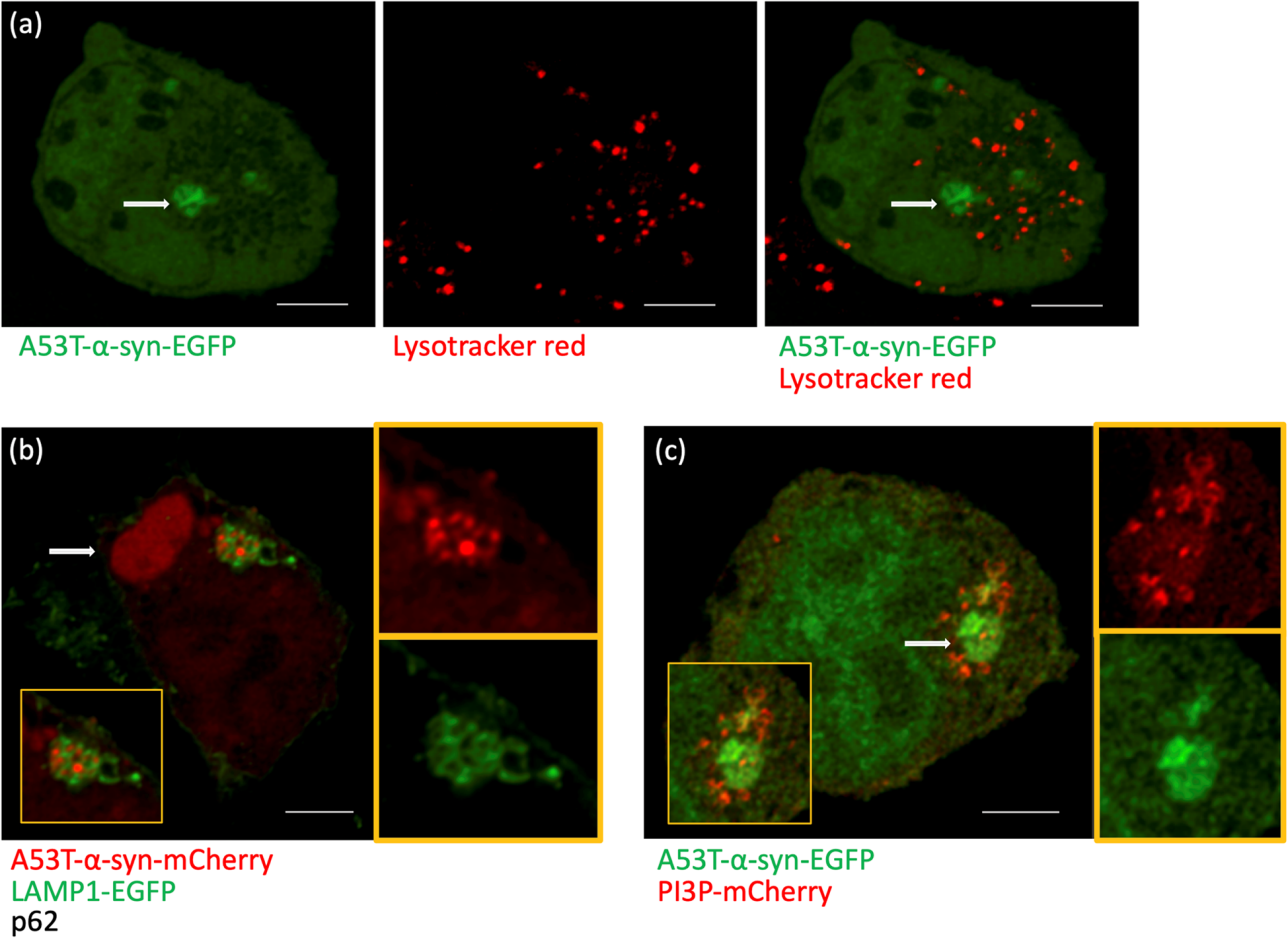
α-Synuclein particles and acidic compartments by light microscopy. **a** Deconvolved confocal images of live HEK293T cells transfected with A53T-α-synuclein (Syn)-EGFP and treated with lysotracker red for 2 h at 37 °C. Arrows show an aggresome. Note the distribution of lysotracker-positive vesicles close to the aggresome and other αSyn aggregates. **b** Deconvolved confocal image of HEK293T cells transfected with A53T-α-Syn-mCherry, the lysosomal marker LAMP1-EGFP and the cargo protein p62 without fluorescent tag. Enlarged insets show a cluster of LAMP1-decorated vesicles colocalizing with aSyn. Arrow shows a large aggresome, so called “p62 body”. **c** Deconvolved confocal image of HEK293T cells transfected with A53T-α-syn-EGFP and a biosensor of the autophagosome lipid phosphatidylinositol 3-phosphate (PI3P) tagged to mCherry. Enlarged insets show the PI3P-positive vesicles around the aggresome (arrow), consistent with the hypothesis that autophagosomes degrade aggresomes. In panels **b**-**c**, the red and green channels of the insets are shown individually next to the merged images. Scale bars, 5 μm. Original data

**Fig. 3:**
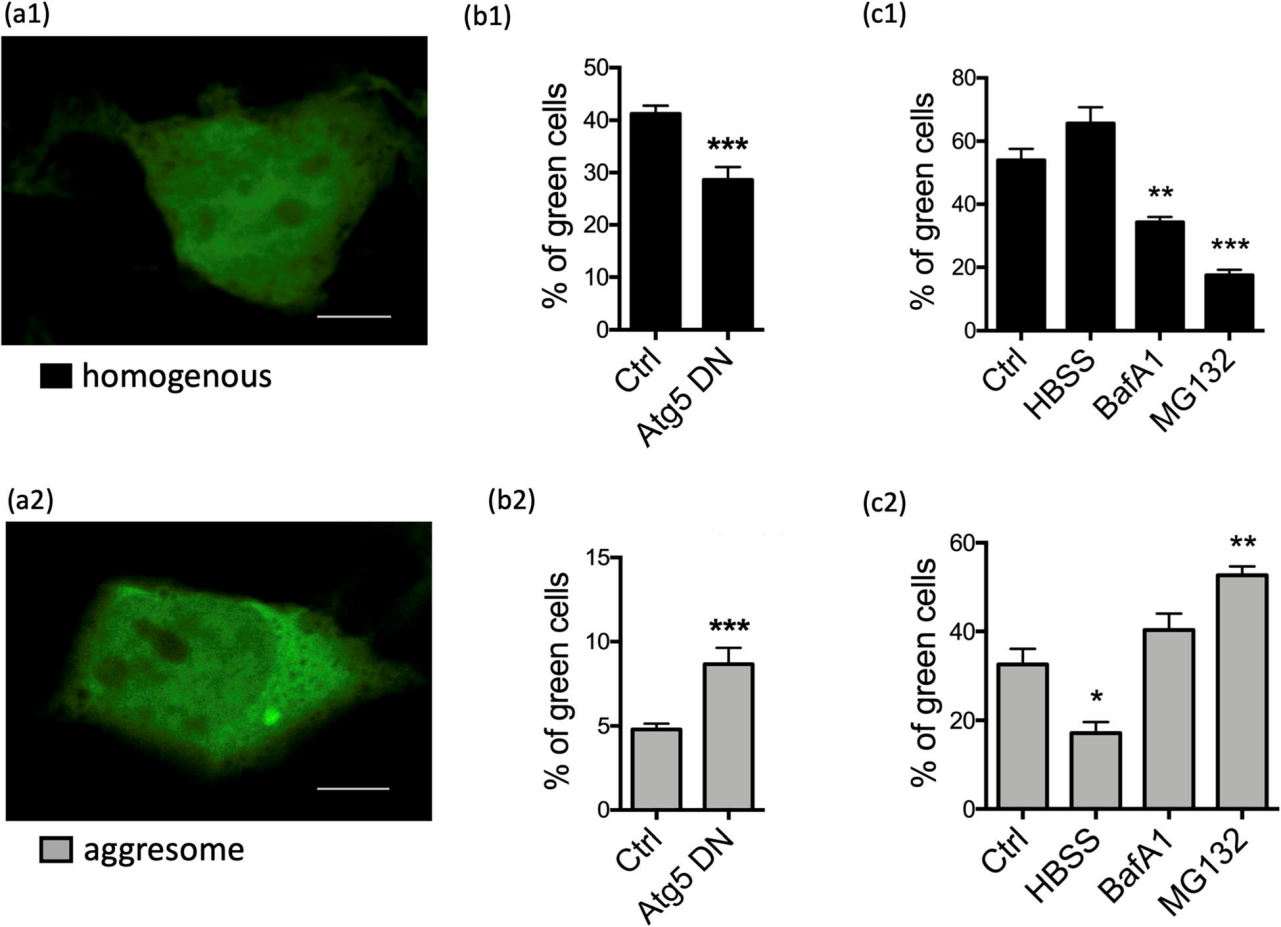
α-Synuclein and autophagy. **a** HEK293 cells were transfected with EGFP-tagged A53T-α-synuclein and manually classified due to the distribution of EGFP. **a1** A representative example of homogenous distribution of EGFP, as the healthy phenotype. **a2** A representative example of a cell containing an aggresome. Scale bars, 5 μm. **b** HEK293 cells were transfected with EGFP-tagged A53T-α-synuclein and the dominant negative version of autophagy-related protein 5 (Atg5). Significances from *t*-test; *n* = 3 independent experiments. **c** HEK293 cells were transfected with EGFP-tagged A53T-α-synuclein and cells were incubated with 0.2 μM Bafilomycin A1 (BafA1) for 4 h to block autophagy or with 5 μM proteasome inhibitor MG132 for 4 h. Starvation was induced by complete medium exchange for Hank’s balanced salts (HBSS) for 4 h. One-way ANOVA was significant, results from *post-hoc* test are indicated (*n* = 3 independent experiments). Graphs represent mean ± SD. *p < 0.05, **p < 0.01, ***p < 0.001. Original data

Cells can degrade aSyn aggregates [44] and they do so mainly by autophagy [48] (Fig. 1b). Autophagy can be stimulated by starvation and by other mechanisms including the Ras-related in brain 7 (Rab7) pathway [49]. The purpose of aggregate transport to the aggresome is to bring together the aggregates and the degradation machinery, i.e. precursors of autophagic vesicles [46, 50]. Autophagosomes are degraded by fusion with lysosomes. This accumulation of autophagosomes and lysosomes around aggresomes and aggregates is illustrated in Fig. 2. Consequently, interfering with dynein functions impairs autophagic degradation of protein aggregates [51]. Overexpression of the cargo protein p62 leads to the formation of large “p62 bodies”, which are to some extent similar to aggresomes [52]. The P62 bodies are also observed with coexpression of p62 and aSyn (Fig. 2b). Autophagy can be induced by starvation (“HBSS” in Fig. 3c1 and c2), leading to more cells without aggregates and a particularly strong reduction of aggresomes rather than that of small dispersed aggregates. The increased aggregate clearance was also observed to be induced by Rab7 and its effector FYCO1 (FYVE and coiled-coil domain 1) [49, 53]. Conversely, blocking the induction of autophagy with a dysfunctional version of the autophagic membrane protein Atg5 leads to a strong increase of aggresomes (Fig. 3b2) and the presence of p62 bodies in neurons of mouse cortex and striatum [52]. The autophagic clearance can also be inhibited by bafilomycin, which blocks the fusion of autophagosomes with lysosomes (Fig. 3c1 and c2). Inhibiting the proteasome (e.g. by MG132) also leads to a strong increase in aggresomes (Fig. 3c2), but this effect is based on the fact that the proteasome clears monomeric synuclein [48]. Thus the proteasome inhibition increases aggregates by increased formation but not by reduced clearance. This reasoning highlights the importance of time-resolved laboratory methods like time-lapse imaging.

Lewy bodies are related to aggresomes [54] and thus signal the occurrence of aggregated proteins. Yet, they are considered part of the cellular defense against protein aggregates and not toxic per se. Aggresomes are the meeting place for aggregates, autophagic membranes, and even lysosomes. Consequently, they are composed not only of densely packed aggregates but also of other cellular components including microtubule-associated proteins [55] and vesicle membranes [56]. The formation of Lewy bodies is in fact a slow maturation process that involves many steps after accumulation of aggregates and vesicles, including posttranslational modifications of aSyn [57]. These findings provide an explanation for the observation in our own work of vesicles positive for the autophagosome lipid phosphatidylinositol 3-phosphate (Fig. 3c) and Rab7 [49].

### Open questions and future perspectives

Several antibodies against aSyn are currently under testing in PD patients. They are well tolerated and have shown a reduction of serum aSyn [58]. This strategy builds on early findings in an aSyn-based mouse model [59, 60], but important questions remain unresolved: (1) What is the ideal epitope against which, antibodies should be targeted? Is it the C-terminus or the N-terminus? Is it monomers, oligomers or fibrils, full-length or truncated aSyn, native or aSyn with posttranslational modifications? (2) Can systemic administration of an antibody deplete aSyn in the brain? Could intrathecal antibodies deplete aSyn in the basal ganglia and brainstem nuclei? (3) How often will antibodies have to be applied and how long will it take to expect an effect on PD symptoms? Can we expect an effect even in manifested PD or do we have to start even earlier? (4) Which PD symptoms are good primary endpoints for aSyn-directed trials? Since dopamine depletion is already quite advanced at the time of diagnosis, and since the dopamine-responsive symptoms are alleviated by current treatments, the non-motor symptoms likely are better candidates. But which non-motor symptoms can be assessed quantitatively and progress in a linear way?

While some of these questions can only be addressed in PD patients, some can be addressed in aSyn-based animal models - certainly in the near future. Which is the best animal model for these investigations? In rodents, many current studies used viral overexpression of aSyn or the administration of pre-formed aSyn fibrils [42, 61–63]. Viral overexpression induces aSyn aggregates, degeneration of dopaminergic neurons and a behavioral phenotype [64, 65]. The validity of aSyn overexpression is supported by the fact that PD can be caused by triplications of the aSyn locus and that polymorphisms in the aSyn promoter region (which affect aSyn expression levels) are important risk factors for sporadic PD. Fibrils can be generated in vitro from recombinant aSyn protein [66], and have been extracted from diseased brains of rodent models or patients [67, 68]. These models have been instrumental to investigate the existence of aSyn strains (see above) and support prion-like properties of aSyn pathology [69]. Work on aSyn aggregation in PD patients is hampered by the fact that brain pathology is available only post mortem. aSyn-based biomarkers in cerebrospinal fluid are under way [70], but still much less developed than for Alzheimer’s disease. aSyn pathology in skin biopsies has shown promise to develop into a biomarker to confirm the diagnosis and possibly even quantify synucleinopathy [71].

## Part 2: Dopamine deficiency

Responsiveness of PD motor symptoms to dopaminergic medication is one of the great medical achievements of the twentieth century [72], constitutes a major diagnostic criterion [1], and highlights the importance of dopaminergic neuron degeneration in PD. Still, many fundamental questions remain unresolved, including the question why dopaminergic neurons in the substantia nigra degenerate in PD even though they are not the only or even the first neurons to show aSyn pathology. Moreover, it has remained enigmatic through which changes in neuronal structure and/or function dopamine deficiency causes PD motor symptoms.

### Selective vulnerability of substantia nigra dopaminergic neurons

Many types of neurons acquire aSyn pathology, but it is mainly dopaminergic neurons in the substantia nigra pars compacta (SNc) that degenerate in PD. Further cell types include noradrenaline and serotonin neurons in the locus coeruleus and raphe [73, 74]. It is therefore plausible to assume that the catecholamine production renders neurons particularly sensitive to neurodegeneration and aSyn toxicity. Indeed, dopamine can form toxic quinones with aSyn [75] and oxidative stress is critical for the propagation of aSyn pathology [8, 64] and neuronal dysfunction [76]. Moreover, catecholamine synthesis is associated with oxidative stress and dopaminergic neurons predominantly degenerate upon systemic exposure to mitochondrial complex I inhibitors such as rotenone [77]. This is consistent with the epidemiological finding that exposure to insecticides constitutes a risk factor for PD [78, 79]. Conversely, aSyn modulates dopaminergic neurotransmission [80], and the induction of aSyn pathology leads to degeneration of dopaminergic neurons [81, 82].

Yet, there are also arguments against the notion that dopamine production is the critical feature that explains degeneration of this neuronal population in PD. The most important argument is the fact that the neighboring population of dopaminergic neurons in the ventral tegmental area degenerates much less in PD patients. Moreover, even among the dopaminergic neurons of the SNc, caudal and ventrolateral subpopulations are affected more strongly [83]. Consequently, additional features of SNc dopaminergic neurons have been tested for their capacity to explain selective vulnerability. These features include firing patterns, specific ion channels and morphological features such as their long and highly branched axons [84–86].

The latter aspect is supported by the notion that dopaminergic axon terminals in the striatum degenerate earlier in PD than their somata in the substantia nigra [87]. The fact that most of the striatal axon terminals are already lost at the time of diagnosis (and at least 50% of substantia nigra dopaminergic neurons) indicates that there is a long time of prodromal PD, consistent with the findings from Lewy pathology. The advanced degeneration of the dopaminergic system at the time of diagnosis means that the dynamic range for further neurodegeneration after diagnosis is small. Classical dopamine-dependent motor symptoms are therefore not good candidates to monitor neurodegeneration in clinical trials with possible neuroprotective strategies. In addition, this notion gives rise to the question of what underlies the qualitative differences between the response to dopaminergic medications in early as compared to advanced PD patients (see below).

### Effects of dopamine deficiency on neuronal activity

**Fig. 4:**
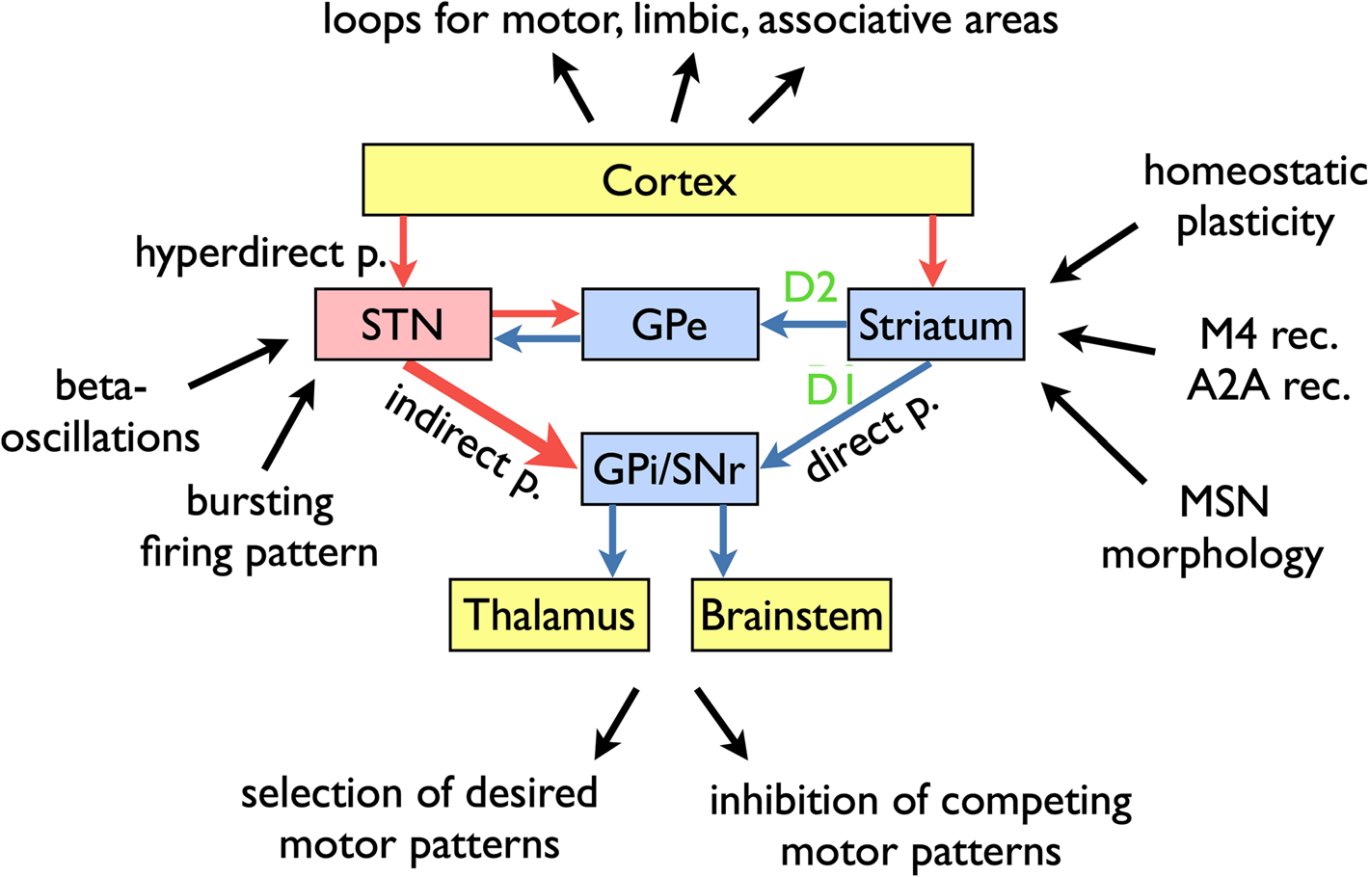
Schematic representation of the basal ganglia circuitry and changes observed in PD patients and models. Distinct loops exist for motor, limbic and associative circuitry. The basal ganglia disinhibit desired motor patterns and inhibit competing motor patterns. The circuitry includes the direct pathway from striatum to GPi/SNr, gated by D1 dopamine receptors, the indirect pathway through GPe and STN, gated by D2 dopamine receptors, and the hyperdirect pathway from cortex to STN. The striatal circuitry is modulated in addition by M4 muscarine and A2A adenosine receptors. In PD, changes occur not only in the firing rate (in particular increased rate of STN firing), but also in the firing patterns, notably the pathological beta-oscillation and increased firing in bursts. Chronic dopamine deficiency leads to the (homeostatic) changes in MSN excitability and morphology. Display of circuitry inspired by Hutchison et al., 2004 [91]. Blue arrows indicate GABAergic projections, and red arrows indicate glutamatergic projections. Abbreviations: p, pathway; rec., receptor; STN, subthalamic nucleus; GPe, globus pallidus pars externa; GPi, globus pallidus pars interna; SNr, substantia nigra pars reticulata; MSN, medium spiny neurons

The dominating model of how dopamine deficiency causes the cardinal motor symptoms was established based on the electrophysiological recordings in 1-methyl-4-phenyl-1,2,3,6-tetrahydropyridine (MPTP)-treated primates [88]. Since its original description, it has been modified and expanded [89, 90]. In the “rate model” [91] (Fig. 4), the striatum functions as the primary entry structure for the basal ganglia and projects to its output nuclei (globus pallidus par interna, GPi, and substantia nigra pars reticulata, SNr) through two pathways: the direct pathway consists of D1 dopamine receptor-bearing striato-pallidal projection neurons; and the indirect pathway is gated by D2 dopamine receptors and projects from striatum to the output nuclei through the globus pallidus pars externa and the subthalamic nucleus (STN). The GABAergic output nuclei project to the thalamus (primarily ventral anterior and ventral lateral nuclei).

The physiological function of this basal ganglia circuitry is to gate motor patterns, i.e., it serves as a “brake” for movements. This brake is too tight in PD and not tight enough in chorea. Our current understanding of basal ganglia functioning includes the following additional aspects:

1. The basal ganglia are not simply a brake but both serve to allow (disinhibit) a desired motor pattern and inhibit competing motor patterns [92].
2. The striatum is not the only input structure for the basal ganglia; there is also a “hyperdirect pathway” to the subthalamic nucleus [91].
3. Movements are not only regulated by the projection from GPi/SNr to the thalamus but also through projections to brainstem nuclei, in particular the pedunculopontine nucleus [90].
4. The basal ganglia are not one big structure for motor control, but consist of several loops that regulate motor, limbic and associative functions [93].

Explaining the therapeutic mechanism of chronic high-frequency stimulation of the STN in PD patients has been a major challenge for the basal ganglia model. One solution to this challenge is to focus on the spatial and temporal patterns of neuronal activity, instead of the overall firing rate. For instance, recordings in PD patients and nonhuman primate models have suggested that a bursting firing pattern is more common and that correlations between neurons are altered [90]. Moreover, oscillations in the basal ganglia circuitry are important determinants of patients’ mobility [91, 94, 95]. Beta oscillations in particular are associated with impaired movement. Their detection can be used to find the best spot for therapeutic STN stimulation. The specific inhibition of these oscillations by phase-locked stimulation can change PD motor symptoms. Importantly, only altered firing patterns and oscillations but not changes in firing rate can explain the emergence of tremor in PD patients. In simple terms, therapeutic deep brain stimulation works mainly by cancelling these pathological firing patterns but not by altering the overall rate of neuronal activity.

### Cellular consequences of dopamine deficiency

Dopaminergic neurons project to virtually all areas of the brain. The densest network of dopaminergic axon terminals is located in the striatum. Spiny projection neurons (or medium spiny neurons, MSNs) are the most abundant cell type in the striatum and thus are most directly affected by dopamine deficiency in PD. From the rate model explained above, we expect D1-MSNs to be less active with dopamine depletion and D2-MSNs to be hyperactive. Yet, MSNs do not remain passive with dopamine depletion. Animal experiments have shown that MSNs rather change their excitability in order to compensate for the loss of dopaminergic innervation. Thus, D1-MSNs increase their electrical excitability and D2-MSNs decrease their electrical excitability after prolonged dopamine depletion. In addition, both types of MSN also change their morphology, showing less complex dendritic arborizations and fewer spines with a reduced number of glutamatergic synapses [96–98]. Similar changes in MSN dendritic arborizations have also been observed in PD patients [99].

These findings – primarily in PD rodent models – highlight the notion that even though it is dopaminergic neurons that degenerate in PD, many more cell types change their functioning in response to dopamine depletion. These “homeostatic” changes are affected by dopaminergic mediation [97, 98] and both the consequences of dopamine depletion and the consequences of excessive dopamine substitution might not be fully reversible. Accordingly, even transient administration of dopamine antagonists can induce lasting movement disorders termed tardive dyskinesias [100].

Moreover, the clinical effects of dopaminergic medications last much longer than what would be expected from pharmacokinetics alone. For levodopa, this “long-duration response” [101] has been partially explained by levodopa storage in dopaminergic terminals. Yet, a long duration response is also seen for dopamine agonists with short half-life [102]. In the classical ELLDOPA trial, the long-duration response - i.e. the difference after 2 weeks without medication in patients either treated for 1 year with levodopa as compared to placebo – made up 50% of the total response to levodopa [103]. It is plausible to assume that the changes in MSN excitability and morphology at least partially underlie the long-duration response to dopamine depletion and dopaminergic medication.

In addition to facilitating movement, dopamine serves as a reward-based teaching signal in many brain areas [104]. Consequently, dopamine deficiency not only leads to the altered steady-state activity, excitability and morphology, but in addition to alterations in long-term potentiation (LTP) and long-term depression (LTD), the neurophysiological correlates of learning and memory. Specifically, dopamine depletion reduces both LTP and LTD induction in the striatum. Short-term and long-term dopamine substitution have different effects, with short-term substitution leading to recovery of LTP and LTD and long-term high-dose substitution leading to a loss of LTD and a loss of LTP depotentiation [105]. These effects could underlie the occurrence of dyskinesias and impulse control disorder in PD patients.

### Future perspectives for translational research

At the timepoint of PD diagnosis, motor symptoms can be readily and continually alleviated by dopaminergic medication whereas in advanced disease, dopaminergic medication may trigger dyskinesia and hallucinations and the duration of the effect is much shorter. The majority of dopaminergic axon terminals are already lost at the timepoint of diagnosis [87]. The qualitative and quantitative differences in the response to dopamine between early and late PD can not only be explained by the progressive loss of dopaminergic terminals. Rather, adaptive and maladaptive changes in the striatum and further basal ganglia nuclei must contribute to these differential effects. We are only beginning to understand the molecular mechanisms behind these changes. Even fundamental biological phenomena such as the adaptation in electrical excitability in response to the changing synaptic inputs are only beginning to be unraveled [106]. Cellular and animal models of dopamine depletion will be critical to address these questions and hold great promise to develop new non-dopaminergic therapies that have the potential to avoid these long-term consequences of dopamine depletion and substitution. In this research, classical toxin-based models of PD (like the 6-OHDA or MPTP models) are used not to study the pathways of dopaminergic neuron degeneration but to determine the consequences of long-term dopamine deficiency. In particular, therapeutic approaches with striatal interneurons [107], M4 muscarine receptors [108] and A2A adenosine receptors [109] have shown promising results.

## Conclusion

PD is a paradigmatic neurodegenerative and movement disorder. Translational studies are aimed into understanding PD pathogenesis and pathophysiology and addressing diverse areas of biophysics, cell biology and neuroscience. In most aspects we have only given a broad overview of current concepts. For a more detailed description we refer to the suggested reading listed below. In many areas, translational research on PD has catalyzed a better understanding of basic biological phenomena, including intrinsically disordered proteins, autophagy, mitochondrial function, homeostatic plasticity and basal ganglia physiology. In some instances, we even had to revise fundamental biological concepts. For instance, the spread of individual aSyn molecules across several synapses and the emerging knowledge about exosomes indicate that neurons are less self-contained entities as stated by the “neuron doctrine” that has dominated neuroscience since the 1890s. Translational research is appealing because of its inherent promise to produce new and refined treatments for human diseases. But it requires a detailed understanding of basic biological phenomena and therefore cannot be conceived without basic sciences.

### Suggested reading

#### Synuclein pathology

Braak H, Ghebremedhin E, Rüb U, Bratzke H, Del Tredici K. Stages in the development of Parkinson’s disease-related pathology. Cell Tissue Res. 2004;318:121–134.

#### Synuclein aggregation and aggregation inhibitors

Pujols J, Peña-Díaz S, Pallarès I, Ventura S. Chemical chaperones as novel drugs for Parkinson’s disease. Trends Mol Med. 2020;26:408–421.

#### Aggregate clearance and the functional significance of Lewy bodies

Lamark T, Johansen T. Aggrephagy: selective disposal of protein aggregates by macroautophagy. Int J Cell Biol. 2012;2012:736905–21.

#### Selective vulnerability of *substantia nigra* dopaminergic neurons

Surmeier DJ, Obeso JA, Halliday GM. Selective neuronal vulnerability in Parkinson disease. Nat Rev. Neurosci. 2017;18:101–113.

#### Effects of dopamine deficiency on neuronal activity

Wichmann T. Changing views of the pathophysiology of Parkinsonism. Mov Disord 2019;34:1130–1143.

#### Cellular consequences of dopamine deficiency

Zhai S, Shen W, Graves SM, Surmeier DJ. Dopaminergic modulation of striatal function and Parkinson’s disease. J Neural Transm. 2019;126: 411–422.

## Data Availability

The raw data and materials used and mentioned in Figs. 2 and 3 are available from the corresponding author on reasonable request.

## Abbreviations

aSyn: alpha-synuclein
Atg5: Autophagy related 5
GPe: Globus pallidus pars externa
GPi: Globus pallidus par interna
LTD: Long-term depression
LTP: Long-term potentiation
MSN: Medium spiny neuron
PD: Parkinson’s disease
Rab7: Ras-related in brain 7
SNc: Substantia nigra pars compacta
SNr: Substantia nigra pars reticulate
STN: Subthalamic nucleus
